# Analysis of high-fidelity simulation effects and their connection with educational practices in early nursing education

**DOI:** 10.1186/s12912-025-03077-x

**Published:** 2025-04-24

**Authors:** Agata Wojcieszek, Anna Kurowska, Aldona Wróbel, Iwona Bodys-Cupak, Alicja Kamińska, Anna Majda

**Affiliations:** Department of Theory and Fundamentals of Nursing, Institute of Nursing and Midwifery Faculty of Health Sciences, Jagiellonian University Medical College, Cracow, Poland

**Keywords:** High-fidelity simulation, Educational practices, Students, Nursing, Satisfaction, Self-confidence

## Abstract

**Background:**

Literature confirms the tangible educational benefits of participating in high-fidelity simulation exercises. However, the final assessment of such sessions is always a combination of the teacher’s actions, the project, the technical infrastructure, and the student. The aim of this study was to evaluate and conduct a comparative analysis of high-fidelity simulation sessions regarding applied educational practices, satisfaction levels, and self-confidence among nursing students at a university located in a major academic center in southern Poland.

**Methods:**

This cross-sectional study was conducted in May and June during the 2021/2022, 2022/2023, and 2023/2024 academic years on a group of 422 first-year undergraduate nursing students. Data from 412 students who participated in high-fidelity simulation sessions were analyzed. The study employed a custom questionnaire, the Educational Practice Questionnaire (EPQ), and the Student Satisfaction and Self-Confidence in Learning Scale (SSCL). The study used Spearman’s correlation coefficient, Kruskal-Wallis test, Dunn’s post-hoc analysis and multivariate linear regression. A significance level of *p* < 0.05 was adopted.

**Results:**

The students rated the attractiveness of the sessions, the development of competencies, and the conducted debriefing relatively highly (average score above 4). Statistically significant differences were found between academic years in the assessment of social competencies acquired (*p* = 0.008) and the evaluation of debriefing elements as a summary method (*p* = 0.009). Students indicated that collaboration (M = 4.81; SD = 0.44) was present in the proposed educational method and considered it the most valuable aspect (M = 4.59; SD = 0.66). A positive correlation (*r* > 0) was noted between the development of knowledge (*r* = 0.389, *p* < 0.001), practical skills (*r* = 0.44, *p* < 0.001), and social skills (*r* = 0.401, *p* < 0.001) and satisfaction. There was also a positive correlation (*r* > 0) between the applied techniques during simulation and the level of self-confidence in the learning process (*p* < 0.05). The applied multiple regression models identified the aspects of the sessions that had a direct and unimpeded impact on nursing students’ sense of satisfaction and self-confidence. These aspects included, among others, active learning and diverse learning methods.

**Conclusions:**

This study confirmed the justification for organizing high-fidelity simulation sessions for nursing students due to the overall benefits for the student (in terms of satisfaction and increased self-confidence), the institution (student satisfaction with the university’s educational offerings), and the profession (high perception of increased nursing competencies).

**Supplementary Information:**

The online version contains supplementary material available at 10.1186/s12912-025-03077-x.

## Background

The continuous and unrestrained development of medicine necessitates the implementation of new learning methods and the simultaneous improvement of those commonly used by teaching staff [1]. One method that has been successfully introduced into the training of future medical personnel is high-fidelity simulation (HFS). The specificity of these sessions relies on creating a teaching room that faithfully replicates the hospital environment, where the patient is replaced by a simulated patient (an actor playing an assigned role) or a simulator. Additionally, the room where the exercises take place is equipped with advanced audio-visual equipment, cameras, and software, allowing the scenario to be conducted and subsequently discussed during the so-called debriefing [2].

HFS is used in the education of future doctors [3], nurses [4], midwives [5], paramedics [6], dentists [7], and even veterinarians [8]. A deeper exploration of the literature regarding the use of HFS in nursing reveals that this method is employed at every stage of education – from undergraduate studies [9] to master’s level education [10]. Furthermore, HFS can be helpful in developing both basic [9] and complex skills, and the planned educational activities enable the acquisition of knowledge that can be applied in the care of patients from various age groups – from pediatrics to geriatrics [9, 11].

There is considerable evidence that the use HFS brings measurable educational benefits. Several available meta-analyses suggest that students actively participating in such sessions acquire and improve skills in a safe environment, where they can make mistakes, learn from them, and not pose a risk to the patient [12–15]. This contributes to an increase in self-efficacy in clinical situations and a reduction in anxiety and fear levels. Additionally, students learn to collaborate in multidisciplinary teams and have the opportunity to develop non-technical skills such as communication and leadership [16].

The use of HFS in nursing education has an intercontinental dimension, as it has been successfully implemented for several years in various regions, including Europe [17], the United States [18], Asia and Africa [19], and Australia [20]. In most cases, researchers report a positive impact of these sessions, such as on the development of skills [4], critical thinking [21], empathy [9], and the opportunity to experience both positive and negative consequences of one’s actions [22]. However, there are studies that provide compelling evidence of the negative effects of HFS, including the occurrence of anxiety [23], stress [24], and even excessive self-confidence [25]. Furthermore, it should be noted that this is a costly and resource-intensive teaching method [25]. Another limitation of HFS is the restricted duration of the sessions, which may prevent students from having the opportunity to repeatedly practice the scenario [26]. Based on the literature review, it can be hypothesized that most student experiences with HFS are related to specialized nursing care [27, 28] rather than the learning of basic nursing skills. Additionally, due to the overrepresentation of studies primarily focused on the development of practical skills during sessions, there is a risk of overlooking the value of therapeutic communication and collaboration. These ambivalent findings provided the research team with the impetus to conduct an evaluation and assess the level of satisfaction with the sessions, especially as this was the first (but not the last) contact for nursing students with HFS. As mentioned earlier, the literature contains publications confirming the positive impact of HFS and general satisfaction with participation in these sessions. However, it should be remembered that the final outcome is always the result of the combined efforts of the teacher, student, course design, and the technical resources used during the sessions.

The value of this study lies in the assessment of the justification for conducting sessions using HFS among nursing students at an early stage of education (first year of studies). Organizing such sessions requires the development of scenarios with an appropriate level of complexity, which, on the one hand, are based on skills acquired in the nursing skills lab, and on the other hand, present an educational challenge without causing cognitive overload. Another strength of the study is the focus on the perception of the obtained educational outcomes and the evaluation of the debriefing process.

The main objective of the study was to present and conduct a comparative analysis of the opinions of the participants regarding high-fidelity simulated HFS sessions, assess the level of satisfaction and self-confidence, and demonstrate the relationship between satisfaction with the sessions and the “competence triangle,” as well as the educational practices applied.

To ensure high-quality simulated sessions, the National League of Nursing (NLN) has outlined key elements to focus on in three simulation areas: simulation design features (e.g., objectives, fidelity, problem-solving, discussion), educational practices (e.g., active learning, collaboration, high expectations, diverse learning methods), and outcomes (e.g., learner satisfaction, critical thinking, and self-confidence) [29]. The tools used in this study evaluate elements from the above simulation areas, such as the achievement of simulation objectives (assessment of educational outcomes, debriefing evaluation – a self-constructed questionnaire), the presence and significance of the applied educational practices, and the outcome of the conducted educational event in terms of determining the level of satisfaction and self-confidence.

After reviewing the literature, the research team hypothesized that students would express general satisfaction with the sessions, satisfaction, self-confidence, and that these sessions would primarily impact the development of practical skills. Among the mentioned educational practices, students would particularly appreciate active learning. The greatest positive impact on the increase in satisfaction and self-confidence after the sessions would be attributed to active learning and collaboration.

## Methods

The study was cross-sectional in nature. The pilot study was conducted during the 2021/2022 academic year. The main study took place during the academic cycles of 2022/2023 and 2023/2024. No changes were made to the research tools after the pilot study, which is why the results from the pilot study were included in the main analysis, forming a comparative analysis. The inclusion criteria for the study were: student status in the nursing program and participation in courses related to basic nursing at the Center for Innovative Medical Education (CIEM).

The study group consisted of first-year full-time nursing bachelor’s students enrolled at a university located in one of the major academic centers in southern Poland. The study employed a convenience sampling method, a form of purposive selection, where units are chosen based on the researcher’s assessment and availability, in contrast to probability sampling, where units are randomly selected from the target population. A total of 436 students were invited to participate in the study, of which 422 completed the questionnaires. Due to significant missing data, the analysis was based on 412 completed research tools. The response rate for the questionnaires was 94%.

The simulated sessions took place in the second semester (in May and June of the aforementioned years) and were preceded by several months of exercises in a low-fidelity skills lab as part of the Basic Nursing course. For the purposes of the simulated sessions, a duo of experienced instructors (including AWo) prepared 7 scenarios, focusing on the consolidation of urinary catheterization skills. The competent judges – members of the research team (AKa, AWr, AKu, IBC, AM) – were then asked to review and identify three scenarios that represented a similar level of difficulty (Appendix 1). Each of the developed scenarios represented a different indication for urinary catheterization: urinary retention after surgery, catheterization for diagnostic purposes, and fluid balance in a patient with circulatory failure.

The scenarios were designed in alignment with the learning outcomes in the areas of knowledge, practical skills, and social skills, as outlined in the curriculum framework for the first year of nursing studies. A checklist was also created for the simulation sessions, which served as an auxiliary tool for the instructor; completing it was helpful for conducting the debriefing (Appendix 2). In addition to performing the procedure (using a sterile, single-use urinary catheterization set while adhering to aseptic and antiseptic principles, according to scenarios involving complex case descriptions), students were required to establish and maintain communication with the patient or their family, as well as communicate effectively within a two-person team. Each of the instructors leading the sessions at the simulation center completed practical training organized by CIEM, during which they acquired knowledge and skills for conducting HFS exercises. The session structure included pre-debriefing, procedure performance, and debriefing.

Students who participated in the sessions at CIEM were divided into 6-person groups. During the HFS sessions, each group worked with 3 scenarios, meaning that students completed a single scenario in 2-person teams. This division ensured that every participant actively engaged in the simulation and had the opportunity to achieve learning outcomes in the areas of knowledge, practical skills, and social skills for their assigned scenario. While two individuals provided patient care, the remaining participants were passive and served as observers. Participation in the HFS was mandatory, forming an integral part of the practical classes in Basic Nursing, but did not involve grading.

Immediately after the completion of the simulation session, which, as mentioned earlier, involved the execution of 3 scenarios, the project leader (AWo) and the research team briefed the students about the general information related to the survey being conducted. They were informed about the anonymous and voluntary nature of the study, as well as the possibility of withdrawing from the study at any point without providing a reason or facing any consequences. Participants who provided informed consent and chose to take part in the study were given printed copies of the research instruments, which also included the Participant Information Sheet, by the research team (AKu, AWr, AKa, IBC). It was ensured that the individuals distributing the forms were not the same as those conducting the sessions. Upon completion, the students were instructed to place the forms in a sealed ballot box located in the CIEM. After the completion of the study, the data were stored in a secure and well-protected location, namely on a password-protected work computer with an encrypted hard drive. Access to the data was restricted solely to the project leader (AWo). The data will be stored for no longer than five years following the completion of the study and data analysis.

The study used diagnostic survey and estimation methods. The diagnostic survey utilized a custom questionnaire, while the estimation method employed standardized scales: the Student Satisfaction and Self-Confidence in Learning Scale (SSCL) and the Educational Practices Questionnaire (EPQ).

- A self-designed questionnaire was used to evaluate the simulated sessions. It assessed the impact of HFS on the development of knowledge, practical, and social skills, as well as opinions on the attractiveness of the sessions and debriefing elements. It contained 16 single-choice questions. Responses were given on a Likert scale (strongly disagree − 1 point, disagree − 2 points, neutral − 3 points, agree − 4 points, strongly agree − 5 points).
- Student Satisfaction and Self-Confidence in Learning Scale (SSCL) - This tool consists of two subscales used to evaluate personal feelings and satisfaction with the simulation. They measure students’ satisfaction with the learning process (five statements) and self-confidence in the learning process (eight statements). The scale includes a total of thirteen statements, each rated on a five-point scale. The scale was authored by Pamela R. Jeffries and Mary Anne Rizzolo and was developed and validated for the Polish version by Katarzyna Studnicka, Danuta Zarzycka, and Jakub Zalewski. The reliability, calculated using Cronbach’s alpha, was 0.87 for satisfaction and 0.84 for self-confidence [30, 31].
- Educational Practices Questionnaire (EPQ) - The authors of the scale are Pamela Jeffries and Mary Rizzolo. This tool consists of four subscales used to assess the application of best educational practices during a simulation scenario. The questionnaire has two parts: one asks about the presence of specific features in the simulation, and the other asks about their importance/relevance to the student. The subscales measure active learning (ten statements), collaboration (two statements), diverse learning methods (two statements), and high expectations (two statements). The scale consists of sixteen statements in total, each rated on a five-point scale. The Polish version of the questionnaire was developed and validated by Katarzyna Zalewska and Danuta Zarzycka. In their publication, the authors of the adaptation used the terms “educational practices” and “educational techniques” interchangeably. The reliability was checked using Cronbach’s alpha coefficient, which was 0.86 for the presence of specific educational practices and 0.91 for the importance of these practices to the respondent [32, 33].

The study, conducted as part of statutory project No. N43/DBS/000253, was approved by the Research Ethics Committee (decision No. 118.6120.28.2022).

Quantitative variables were analyzed by calculating descriptive statistics such as mean, standard deviation, median, quartiles, and minimum and maximum values. Correlations between quantitative variables were analyzed using Spearman’s correlation coefficient. Comparisons of quantitative variable values in three or more groups were made using the Kruskal-Wallis test, and, if statistically significant differences between groups were detected, Dunn’s post-hoc test was used. To assess the influence of selected independent variables on the dependent variables, multivariate linear regression was applied. A significance level of 0.05 was adopted for the analysis. The results were processed using R software, version 4.4.1 [34].

## Results

The data obtained from 412 students were analyzed. A significantly higher participation of women was noted (*N* = 382; 92.72%) compared to men (*N* = 30; 7.28%). The students ranged in age from 20 to 22 years.

The average score for the overall attractiveness of the HFS sessions ranged from 4.59 to 4.69 points (out of 5 possible). Regarding the knowledge gained during the HFS sessions, the students’ scores ranged from 4.46 to 4.5, for practical skills from 4.46 to 4.58, and for social skills from 4.11 to 4.44, with the evaluation of interpersonal skill development being significantly higher in 2024 than in 2022 and 2023.

The students’ ratings for the debriefing as part of the simulation, indicating the correctness of the performed elements, were similar across all three groups, ranging from 4.86 to 4.92 points. In contrast, the results for the function of debriefing as part of the session, indicating elements that needed improvement, ranged from 4.76 to 4.83 points. The results for pointing out methods for correcting mistakes during the debriefing ranged from 4.74 to 4.87. The final element of the evaluation, the assessment of debriefing as an appropriate method for summarizing the sessions, received an average score ranging from 4.75 to 4.92, with this rating being noticeably higher in the 2023/2024 and 2021/2022 academic years than in 2022/2023 (Table 1).

**Table 1:** Summary of nursing students’ opinions on the attractiveness of the sessions, acquired competencies, and debriefing evaluation

Variable	Annual	*N*	M	SD	Me	Min	Max	Q1	Q3	*p*
Assessment of the attractiveness of classes	2022	126	4.59	0.67	5	1	5	4	5	*p* = 0.411
	2023	153	4.61	0.59	5	3	5	4	5	
	2024	133	4.69	0.52	5	3	5	4	5	
	Total	412	4.63	0.6	5	1	5	4	5	
Assessment of knowledge development	2022	126	4.46	0.76	5	1	5	4	5	*p* = 0.938
	2023	153	4.48	0.64	5	2	5	4	5	
	2024	133	4.5	0.62	5	2	5	4	5	
	Total	412	4.48	0.67	5	1	5	4	5	
Assessment of practical skills development	2022	126	4.52	0.7	5	1	5	4	5	*p* = 0.304
	2023	153	4.46	0.7	5	2	5	4	5	
	2024	133	4.58	0.64	5	2	5	4	5	
	Total	412	4.51	0.68	5	1	5	4	5	
Assessment of social skills development	2022	126	4.13	0.95	4	1	5	4	5	*p* = 0.008 * 2024 > 2022,2023
	2023	153	4.11	1	4	1	5	4	5	
	2024	133	4.44	0.74	5	2	5	4	5	
	Total	412	4.22	0.92	4	1	5	4	5	
Assessment of debriefing in the context of indicating correctly performed elements	2022	126	4.88	0.35	5	3	5	5	5	*p* = 0.091
	2023	153	4.86	0.39	5	3	5	5	5	
	2024	133	4.92	0.34	5	3	5	5	5	
	Total	412	4.89	0.36	5	3	5	5	5	
Assessment of debrifing in the context of identifying elements for improvement	2022	126	4.83	0.53	5	2	5	5	5	*p* = 0.317
	2023	153	4.76	0.67	5	1	5	5	5	
	2024	133	4.8	0.73	5	1	5	5	5	
	Total	412	4.79	0.65	5	1	5	5	5	
Assessment of the indication of error correction methods in debriefing	2022	126	4.87	0.44	5	2	5	5	5	*p* = 0.133
	2023	153	4.74	0.72	5	1	5	5	5	
	2024	133	4.78	0.75	5	1	5	5	5	
	Total	412	4.79	0.66	5	1	5	5	5	
Assessment of debriefing elements conducive to the debriefing of classes	2022	126	4.91	0.31	5	3	5	5	5	*p* = 0.009 * 2024,2022 > 2023
	2023	153	4.75	0.72	5	1	5	5	5	
	2024	133	4.92	0.32	5	3	5	5	5	
	Total	412	4.85	0.51	5	1	5	5	5	

p - Kruskal-Wallis test + post-hoc analysis (Dunn’s test), N - number of observations, M-mean, SD-standard deviation, Me-median, Min-minimum, Max-maximum, Q1-quartile 1 and Q3-quartile 3. Source: authors’ analysis

The mean score for the presence of active learning (M = 4.43; SD = 0.51) and diverse learning methods (M = 4.32; SD = 0.74) indicated that these educational practices were present during the simulation. The mean score for the presence of collaboration (M = 4.8; SD = 0.44) and high expectations (M = 4.6; SD = 0.58) suggested that these elements were clearly present (rounded to 5) in the proposed educational method (Table 2).

The mean score for the importance of active learning (M = 4.36; SD = 0.57), diverse learning methods (M = 4.26; SD = 0.80), and aspects related to high expectations (M = 4.49; SD = 0.69) were considered important by the respondents in the proposed educational method. The mean score for collaboration was M = 4.59, SD = 0.66, making this aspect critically significant for the respondents (Table 2).

**Table 2:** Descriptive statistics illustrating respondents’ answers regarding the presence and importance of specific educational techniques

EPQ - attendance	*N*	ND	M	SD	Me	Min	Max	Q1	Q3
Active learning	412	0	4.43	0.51	4.5	2.0	5	4.1	4.81
Collaboration	411	1	4.81	0.44	5.0	3.0	5	5.0	5.00
Learning diversity	408	4	4.32	0.74	4.5	1.5	5	4.0	5.00
High expectation	412	0	4.60	0.58	5.0	2.0	5	4.5	5.00

EPQ - importance	N	ND	M	SD	Me	Min	Max	Q1	Q3
Active learning	412	0	4.36	0.57	4.44	1.1	5	4.0	4.8
Collaboration	412	0	4.59	0.66	5.00	1.0	5	4.5	5.0
Learning diversity	408	4	4.26	0.80	4.50	1.0	5	4.0	5.0
High expectation	412	0	4.49	0.69	5.00	1.0	5	4.0	5.0

N-number of observations, ND-no data, M-mean, SD-standard deviation, Me-median, Min-minimum, Max-maximum, Q1-quartile 1 and Q3-quartile 3. Source: authors’ analysis

The average score on the learning process satisfaction scale was 22.08 points, which equals 4.42 points per question (rounded to 4). This indicated that the respondents expressed satisfaction with the learning process (Table 3).

The average score on the self-confidence scale in the learning process was 34.9 points, which equals 4.36 points per question (rounded to 4). Thus, the respondents expressed confidence in the learning process (Table 3).

**Table 3:** Summary of the responses from the surveyed students regarding satisfaction and self-confidence after participating in simulation session

SSCL	Point range	*N*	ND	M	SD	Average per question	Me	Min	Max	Q1	Q3
Learning satisfaction	5–25	412	0	22.08	2.88	4.42	23	6	25	20	25
Confidence in learning	8–40	412	0	34.90	3.94	4.36	35	14	40	32	38

N-number of observations, ND-no data, M-mean, SD-standard deviation, Me-median, Min-minimum, Max-maximum, Q1-quartile 1 and Q3-quartile 3,SSCL-Student Satisfaction and Self-Confidence in Learning Scale

Source: authors’ analysis

Statistically significant differences were noted between the groups in terms of satisfaction level (*p* = 0.001) and self-confidence (*p* = 0.017) during the HFS process (Table 4).

**Table 4:** Comparison of the surveyed cohorts in terms of satisfaction and self-confidence after participating in simulation sessions

SSCL	Annual	*N*	M	SD	Me	Min	Max	Q1	Q3	*p*
Learning satisfaction	2022	126	22.02	2.82	23	10	25	20	24	*p* = 0.001 2024 > 2022,2023
	2023	153	21.56	3.05	22	6	25	20	24	
	2024	133	22.75	2.61	24	15	25	21	25	
Confidence in learning	2022	126	34.79	3.76	35	24	40	32.25	38	*p* = 0.017 2024 > 2023, 2022
	2023	153	34.3	4.17	34	14	40	32	37	
	2024	133	35.69	3.73	36	27	40	33	39	

p - Kruskal-Wallis test + post-hoc analysis (Dunn’s test), N-number of observations, M-mean, SD-standard deviation, Me-median, Min-minimum, Max-maximum, Q1-quartile 1 and Q3-quartile 3, SSCL-student Satisfaction and Self-Confidence in Learning ScaleSource: authors’ analysis

The result of the learning process satisfaction scale was significantly (*p* < 0.05) and positively (*r* > 0) correlated with the assessment of the development of knowledge, practical skills, and social skills (Table 5).

**Table 5:** The relationship between the respondents’ opinions on the perception of learning outcomes and satisfaction with the learning process

Variable	Learning satisfaction
	Spearman’s correlation coefficient
Assessment of knowledge development	*r* = 0.389, *p* < 0.001
Assessment of practical skills development	*r* = 0.44, *p* < 0.001
Assessment of social skills development	*r* = 0.401, *p* < 0.001

p-statistical value; r-Spearman’s rank correlation coefficient

Source: authors’ analysis

The self-confidence result in the learning process was significantly (*p* < 0.05) and positively (*r* > 0) correlated with the score on the scale of active learning presence, collaboration, diverse learning methods, and expectations (Table 6).

**Table 6:** The relationship between the techniques used during the simulation and self-confidence in the learning process

Variable	Confidence in learning
	Spearman’s correlation coefficient
Active learning–attendance	*r* = 0.589, *p* < 0.001 *
Collaboration–attendance	*r* = 0.266, *p* < 0.001 *
Learning diversity–attendance	*r* = 0.51, *p* < 0.001 *
High expectations - attendance	*r* = 0.502, *p* < 0.001 *

p-statistical value; r-Spearman’s rank correlation coefficient

Source: authors’ analysis

The results of the univariate regression analysis revealed that each additional point obtained in the evaluation of various aspects of the simulation led to a higher score on the satisfaction scale regarding the learning process. The greatest increase in satisfaction was observed in the presence (increase of 3.7 points) and significance (2.575 points) of active learning, as well as the inclusion of expectations (2.315 points) in the simulation design. The smallest increase was noted in the role of debriefing as a tool for analyzing methods of error correction (0.708 points) and in the scale of the significance of collaboration (1.005 points) (Table 6).

The multivariable regression models indicated that each additional point in the evaluation of the attractiveness of the sessions, development of practical skills, presence of active learning, diverse teaching methods, and significance of expectations significantly increased the nursing students’ satisfaction scores. The greatest increase in satisfaction was noted in the area of active learning (increase in confidence by 2.111 points), while the smallest increase was observed with regard to the importance of expectations (0.448 points) (Table 7).

**Table 7:** Results of univariate and multivariate regression analysis presenting the relationship between session attractiveness, perception of acquired competencies, educational practices, and participants’ confidence

Variables	Single-factor models				Multi-factor models			
	Value	95%CI		*p*	Value	95%CI		*p*
Assessment of the attractiveness of classes	2.151	1.546	2.755	< 0.001*	0.33	-0.256	0.915	0.271
Assessment of knowledge development	1.748	1.206	2.291	< 0.001*	-0.171	-0.735	0.392	0.552
Assessment of practical skills development	2.109	1.588	2.63	< 0.001*	0.409	-0.195	1.014	0.185
Assessment of social skills development	1.491	1.101	1.882	< 0.001*	0.216	-0.189	0.62	0.296
Assessment of debriefing in the context of indicating correctly performed elements	2.428	1.398	3.457	< 0.001*	1.02	0.03	2.011	0.044*
Assessment of debrifing in the context of identifying elements for improvement	0.331	-0.256	0.917	0.27	-	-	-	-
Assessment of the indication of error correction methods in debriefing	0.648	0.075	1.222	0.027*	-0.417	-1.056	0.223	0.202
Assessment of debriefing elements conducive to the debriefing of classes	1.379	0.644	2.113	< 0.001*	0.303	-0.565	1.171	0.494
Active learning - attendance	4.457	3.845	5.07	< 0.001*	2.066	1.035	3.097	< 0.001*
Collaboration - attendance	2.377	1.534	3.219	< 0.001*	-0.622	-1.493	0.249	0.163
Learning diversity - attendance	2.621	2.173	3.069	< 0.001*	1.034	0.403	1.665	0.001*
High expectation - attendance	3.533	2.975	4.091	< 0.001*	1.333	0.586	2.08	0.001*
Active learning - importance	3.209	2.612	3.806	< 0.001*	0.241	-0.705	1.188	0.618
Collaboration - importance	1.477	0.921	2.034	< 0.001*	0.288	-0.317	0.893	0.352
Learning diversity - importance	2.054	1.621	2.487	< 0.001*	0.172	-0.428	0.773	0.574
High expectation - importance	2.363	1.86	2.866	< 0.001*	0.08	-0.582	0.742	0.813

CI - confidence interval, *statistically significant relationship (*p* < 0.05)

The results of the univariate regression analysis indicate that each additional point obtained in the areas of session attractiveness, perception of acquired competencies, evaluation of certain elements of debriefing, and the presence and significance of applied educational practices leads to an increase in the confidence score in the learning process. The largest increase in confidence was observed in the presence of active learning (4.457 points) and high expectations (3.533 points), while the smallest increase was related to the evaluation of error correction methods in debriefing (0.648 points) and its role in summarizing the sessions (1.379 points) (Table 7).

The results of the multivariate regression model revealed that each additional point obtained in identifying correct elements during scenario execution (increase in confidence by 1.02 points), presence of active learning (2.066 points), diverse learning methods (1.034 points), and high expectations (1.333 points) led to an increase in the confidence score in the learning process (Table 7).

## Discussion

The design of courses, particularly those utilizing HFS, carries significant responsibility, as the creators of simulation scenarios, we shape a segment of reality that influences the future experiences of students. The sessions conducted using HFS were highly rated by the participating students, as each assessment element received over 4 points out of a possible 5. Both the attractiveness of the sessions and their impact on learning in the cognitive, affective, and psychomotor domains were positively evaluated by the students. It is worth noting that students who participated in the third year of evaluation rated the development of social skills significantly higher, as confirmed by the post-hoc Dunn analysis. This may be due to the fact that in the academic year 2023/2024, no classes were conducted in which students had contact with a standardized patient - a trained individual who could realistically portray a patient in a consistent and standardized manner.

As mentioned in the methodology section, HFS sessions were preceded by several months of exercises in low-fidelity labs, including work with mannequins and models. Although practical skills can be developed in such settings, the application of therapeutic communication is limited by the acting skills of the instructor or the peer group. In the case of first-year students, the focus often lies on the correct performance of procedures. Only after practicing and mastering practical skills can students focus on the communicative and care aspects of the situation [22]. In our scenarios, we designed opportunities to apply therapeutic communication in the context of a patient who is reluctant to continue treatment and care, a patient with excessive health anxiety, and a demanding and entitled female patient. Participation in the simulation allowed students to establish and maintain contact with the patient in a safe environment, with this being the first such opportunity for students from the 2023/2024 teaching cycle.

Debriefing, as an appropriate method of concluding the sessions, was significantly better rated by students surveyed in the academic years 2021/2022 and 2023/2024. It is difficult to definitively determine the reasons for this, but considering that the sessions in the different years were conducted according to the adopted model and consistent procedural framework, the poorer ratings may have been influenced by personality factors of the students, perceptions of the instructor, or other elements that unfortunately were not examined. A thorough analysis of the data presented in Table 1 reveals that the 2023/2024 cohort recorded the lowest average scores for each aspect of debriefing. However, it is worth noting that these scores were still high (above 4.5 points out of a possible 5). Numerous studies indicate that debriefing is the most important element of simulation, as it allows instructors and students to analyze the experience of the simulated case, understand the reasoning behind a given clinical judgment, and identify the desired way to solve the problem. The results obtained are satisfactory in the opinion of the research team, as they support the belief that the three-phase debriefing technique, known as “Debriefing with Good Judgment,” consisting of the reaction, analysis, and summary (RAS) phases, was beneficial [35].

In the presented study, students had the opportunity to actively participate in the sessions and benefit from diverse teaching methods, which proved to be highly valuable for them. In the literature, active participation is understood as a purposeful, collaborative, knowledge-based action contributing to the achievement of a common goal. In the case of nursing students, this goal should focus on providing holistic care in response to the dynamic state of the patient [36]. An interesting study, with a similar methodology (using the NLN/Jeffries Nursing Education Simulation Framework), was conducted in Oman with a group of 370 nursing students. Approximately 77.5% of nursing students believed that simulation had an additive impact on collaboration, 80.9% stated that simulation facilitated active learning, and 81.6% of students asserted that HFS provided an opportunity for diverse learning. The highest percentage, 84.8%, rated simulation as creating high expectations regarding patient care [37]. The role of active learning was emphasized in research by Blanié A. et al., involving a group of 104 anesthesiology residents participating in a simulation session. It was found that active participation in HFS was associated with a better evaluation of educational benefits, such as increased knowledge, non-technical skills, and satisfaction, compared to passive observation [38]. Haddeland highlights the significant interest among students in active participation in HFS, which may not be feasible with a large group of students participating in the simulation session [39]. However, there are differing scientific reports providing evidence that both observation and active participation were associated with similar, desired educational outcomes [40]. This could be due to the fact that individuals in the role of observers have the opportunity for a more objective judgment and can analyze the situation without emotional bias, thereby gaining benefits for themselves. Arbitrarily, it can be concluded that it is worthwhile to design HFS sessions in such a way that participants can learn both through action and observation.

The students surveyed indicated that the simulation session significantly met their educational needs, which varied depending on their personal characteristics and preferences. Furthermore, high expectations from the facilitator greatly stimulated the students to utilize their own resources to achieve the simulation goals. Cooperation among participants was strongly present in the simulations, and this aspect was considered the most important by the students. This is an interesting issue, as educational activities typically emphasize the development of knowledge or skill acquisition, while collaboration or soft skills are rarely the direct focus of educational interventions [22]. This may be because data related to manual/technical performance is more accessible than data regarding interactions between students and patients. It is valuable that during the HFS sessions, students were able to work in groups, solve problems collaboratively, and develop cooperation in a safe environment. A systematic review conducted by Li Y. analyzed the impact of HFS on various aspects of education in a group of undergraduate nursing students [41]. In this meta-analysis, data from six studies (with a total of 734 participants) confirmed that HFS is more effective in promoting collaboration compared to other teaching methods. Similar findings, based on student experiences, exploring cooperation in simulation sessions, stem from experimental studies [42] and qualitative analyses of student feedback [43]. In our study, first-year nursing students had the opportunity to work in a two-person team, collaborating within a peer group. This can be a valuable lesson for future stages of their education, where interdisciplinary teamwork will be essential.

The proposed educational method provided first-year students with a source of satisfaction and self-confidence, with a statistically significant difference indicating the greatest intensification of these variables among students surveyed in the 2023/2024 academic year. Organizing activities at such an early stage of nursing education was a challenge due to the fact that HFS exercises were preceded by sessions conducted in a low-fidelity laboratory. Developing simulation scenarios requires focusing on learning outcomes and ensuring appropriate stimulation that, on one hand, takes into account the limited resources and capabilities of the student (depending on the stage of education) and, on the other hand, allows for their development. Despite the variety of simulation environments and methodologies, there is general consensus that participation in sessions utilizing advanced mannequins and a clinical environment simulation is attractive to students [27, 28, 44]. The research process revealed a positive correlation between satisfaction and the “competency triangle”, meaning that a better opinion regarding the achieved learning outcomes (knowledge, practical and social skills) generated greater satisfaction with the HFS sessions. It should be noted that the concept of satisfaction in the context of HFS teaching should be understood as the opportunity to implement a scenario according to one’s knowledge of bladder catheterization, understanding indications, contraindications, and the procedure itself, instrumental skills, using therapeutic communication, and considering subjectivity in relationships. All of this should coexist with a positive attitude toward the applied teaching strategy. Furthermore, the use of active learning techniques, collaboration, diverse learning methods, and expectations in the simulation was associated with a higher level of self-confidence. In the context of simulation, it can be said that the coexistence of these elements within the simulation contributed to students’ sense of having the resources necessary to solve a complex task during the simulation process.

Similar results were obtained in Khasawneh’s study, which confirmed the existence of a strong correlation between the simulation design, educational practices, and nursing students’ satisfaction and self-confidence [37]. The research process conducted by Olaussen C., with a group of 187 Norwegian students, showed that active learning can increase both students’ satisfaction with the learning activity and their self-confidence in handling a simulated patient situation, and educators should be particularly interested in ensuring opportunities for active participation in the learning process [45]. In their study, the average scores for self-confidence and satisfaction were 4.16 and 4.57, respectively, while in this study, they were 4.36 and 4.42. Another study initiated by Gabbouj among 110 students reported an overall satisfaction score of M = 21, SD = 3.5 (in our study, M = 22.08; SD = 2.88) and self-confidence of M = 33.8, SD = 4.7 (M = 34.90; SD = 3.94) [46]. The results of the aforementioned studies were consistent with those obtained in our own study. In addition to performing correlation tests aimed at identifying relationships between variables, we decided to deepen the analysis using multifactorial regression. The application of this statistical method aimed to provide answers to the question of how educational practices, the evaluation of class attractiveness, the perception of acquired competencies, and the evaluation of debriefing elements affect the level of satisfaction and self-confidence of the students surveyed. The results of the multifactorial regression analysis revealed that organizing attractive classes developing practical skills with active learning and diverse learning methods had a direct and undisturbed impact on satisfaction with the simulation sessions. Moreover, designing activities that enabled active learning, based on ambitious scenarios and diverse teaching methods, as well as emphasizing correctly performed elements during debriefing, significantly stimulated first-year nursing students’ self-confidence. Regarding many other aspects of the classes, a significant relationship was found in the unifactorial analysis but not in the multifactorial regression, which in theory means that, for example, collaboration influenced satisfaction or self-confidence, but only indirectly. This explains the lack of dependency once factors directly influencing these outcomes were accounted for. Therefore, our hypothesis stating the dominant role of active learning and collaboration in achieving satisfaction and self-confidence was only partially confirmed.

This study has several strengths. Firstly, it was a time-consuming 3-year process of data collection on simulation-based teaching with HFS among nursing students. As previously mentioned, first-year students are rarely subjected to studies in simulation centers. In Poland, HFS is already present in medical education, but it remains an area that requires further reflection to maximize educational outcomes. When designing this study, we used well-established research tools specific to simulation, developed by the NLN, which were successfully adapted to the Polish context. The study involved a relatively large group of students (over 400) from three consecutive academic cycles, which enhanced the reliability of the assessment of simulation sessions. Additionally, the courses were designed in such a way that every student actively participated in the simulation and had the opportunity to experience and evaluate the planned educational intervention.

However, our study also had several limitations – a limited scope (the study was conducted within a single university), the inability to generalize the results, and the absence of causal relationships due to the cross-sectional nature of the study. Furthermore, the use of a non-experimental, correlational design may only partially reflect students’ experiences with simulation-based education. The non-random sampling method, based on the convenience and proximity of participants, may limit the generalizability and the ability to draw conclusions about the broader population. Another significant limitation of the study was the lack of a control group with different teaching methods, which made it difficult to assess the effectiveness of simulation in comparison to other educational approaches. In the future, it would be valuable to complement the analysis of results by conducting similar studies after sessions using other modalities – it may turn out that each new educational experience leads to increased satisfaction due to the so-called freshness effect or the lack of a broader perspective. A valuable addition would also be the assessment of knowledge retention and its application in the clinical environment, as well as the evaluation of simulation-based courses after the second or third year of undergraduate or master’s studies (longitudinal studies). Finally, supplementing the quantitative study with an in-depth qualitative investigation within a methodological triangulation framework could provide valuable insights that cannot be captured through survey-based research.

## Conclusions

1. Participation in high-fidelity simulation (HFS) sessions served as a source of satisfaction, contentment, and increased self-confidence for first-year nursing students. This resulted from the high evaluation of the attractiveness of the sessions, positive perceptions of acquired practical skills, the application of active learning, and the use of diverse learning methods and scenarios, which facilitated the maximization of educational outcomes. Additionally, significant importance was placed on fostering students’ sense of self-efficacy by emphasizing correctly performed elements of the scenario during the debriefing.
2. The conducted research process confirmed that organizing high-fidelity simulation sessions for nursing students, even at the early stages of their education, is beneficial due to the overall advantages, including those for the student (in the form of satisfaction and increased self-confidence), for the institution (student satisfaction with the educational offerings), and for the profession (a high perception of increased nursing competencies, not only practical but also social).

## Electronic supplementary material

Below is the link to the electronic supplementary material.

Supplementary Material 1

Supplementary Material 2

## Data Availability

Data is provided within the manuscript or supplementary information files.

## Abbreviations

Aka: Alicja Kamińska
AKu: Anna Kurowska
AM: Anna Majda
AWo: Agata Wojcieszek
AWr: Aldona Wróbel
CIEM: Centre for innovative medical education
EPQ: Educational practices questionnaire
HFS: High-fidelity simulation
IBC: Iwona Bodys-Cupak
NLN: National league of nursing
SSCL: Student satisfaction and self-confidence in learning scale
