# Supplementary material for: Analysis of high-fidelity simulation effects and their connection with educational practices in early nursing education
Source: BMC Nurs. 2025 Apr 24;24:457. doi: 10.1186/s12912-025-03077-x (PMC12023673; doi:10.1186/s12912-025-03077-x)
Supplement: Supplementary file 1 — Supplementary Material 1 [file 12912_2025_3077_MOESM1_ESM.docx]

Simulation scenario no. 1

| Keep it a secret from the training participants.  Knowledge of the scenario reduces the satisfaction of solving the task | | |
| --- | --- | --- |
| Objectives – to be discussed at debriefing | Technical:  - correct insertion of a catheter into the urinary bladder,  - recognition of urinary retention on the day of surgery | Soft skills:  - communicating with an agitated patient  - communicating in a team |
| Title of the simulation scenario | Urinary retention after surgery | |
| Authors | Agata Wojcieszek  Kinga Kołodziej | |
| Contact | [Agata.wojcieszek@uj.edu.pl](mailto:Agata.wojcieszek@uj.edu.pl) | |
| Diagnosis | Urinary retention after surgery | |
| Case description | Patient Jan B. is on day "0" after the surgery. 8 hours have passed since the surgical intervention. The patient experiences pain in the lower abdomen. He is nervous and very afraid. He read online sources of knowledge and it was written there that such pain after the procedure means complications. Using the bell, he calls you to the room. | |
| Team composition | Instructor – plays the role of a nervous patient, supervises the course of the scenario  The rest of the team – nursing students who take part in the scenario | |
| Information for participants | - | Team (roles): We appoint a team leader. |
| Preparing the mannequin | Simulator of a man in a hospital bed wearing a long shirt tied at the back. | |
| Room | Patient's room in a hospital ward | |
| Equipment List | Set for single-use urinary bladder catheterization:  sterile single-use catheter (2 pcs.), sterile gripping tool, sterile single-use gloves (2 pcs.), non-sterile single-use gloves, sterile single-use drape, sterile gauzes (minimum 6 pcs.), anesthetic-lubricant material e.g. 2% lidocaine, antiseptic fluid e.g. Skinsept Mucosa, kidney bowls, treatment tray/trolley, screen, medical waste bags  - Pean for clamping the catheter and gauze  - Equipment for urine provocation (bowl, basin) | |
| Initial parameters  - simulator | At the beginning: 18 breaths/min; SpO2 - 97% Temp. 36.7°C; HR: 76 bpm, regular, well-tensed; RR 125/80 mmHg. Patient groans, abdomen is distended, painful  After 5 minutes: 21 breaths/min; HR: 101 bpm regular, well-tensed; CTK 130/80 mmHg, Patient groans, abdomen is distended, painful | |
| Interview | The patient reports increasing pain in his lower abdomen. He feels a great pressure on his bladder. He would like to urinate, but has trouble doing so. He has read that abdominal pain after surgery can mean something bad - he is terrified by the vision of dialysis, cancer, peritonitis, etc. | |
| Progress of the scenario | 1. Participants enter the room where the patient is  2. Conversation with the patient – ​​the patient reports lower abdominal pain  3. Diagnosis of urinary retention  4. Provocation of diuresis – no urine flow  5. Obtaining consent to perform catheterization  6. Decision to insert a Foley catheter (the bladder contains more than 1500 ml of urine)  7. Clamping the catheter after exceeding 600-800 ml of urine  8. Drainage of urine  9. Removal of the catheter  10. Documentation | |
| Necessary props | Standard equipment of the patient room, cardiac monitor, bladder catheterization kit, urine provocation equipment | |

Simulation scenario no. 2

| Keep it a secret from the training participants.  Knowledge of the scenario reduces the satisfaction of solving the task | | |
| --- | --- | --- |
| Objectives – to be discussed at debriefing | Technical:  - catheterization of the female urinary bladder  - collection of urine for a general examination | Soft skills:  - communicating with a patient with a claim  - communicating within a team |
| Title of the simulation scenario | Bladder catheterization for diagnostic indications | |
| Authors | Agata Wojcieszek  Kinga Kołodziej | |
| Contact | [agata.wojcieszek@uj.edu.pl](mailto:agata.wojcieszek@uj.edu.pl) | |
| Diagnosis | Bladder catheterization for diagnostic indications | |
| Case description | It is 6:00 a.m. You have received an urgent referral from a doctor to collect urine for a general examination of the patient Anna C. (age 35). The day before yesterday in the evening the patient started menstruating. Yesterday an attempt was made to collect urine after previously inserting a tampon into the vagina, but the result was questionable. The patient is nervous. Another urine collection is required, and yesterday she was assured that a tampon would be sufficient. In addition, she is woken up every day at 6:00 a.m., which prevents her from getting enough sleep. | |
| Team composition | Instructor – plays the role of a nervous patient, supervises the course of the scenario  The rest of the team – nursing students who take part in the scenario | |
| Information for participants | - | Team (roles): we appoint a team leader of 2 people |
| Preparing the mannequin | A woman simulator in a hospital bed wearing pajamas | |
| Room | Patient's room in a hospital ward | |
| Equipment List | Set for single-use urinary bladder catheterization:  sterile single-use catheter (2 pcs.), sterile gripping tool, sterile single-use gloves (2 pcs.), non-sterile single-use gloves, sterile single-use drape, sterile gauzes (minimum 6 pcs.), anesthetic-lubricant material e.g. 2% lidocaine, antiseptic fluid e.g. Skinsept Mucosa, kidney bowls, buttock cushion, treatment tray/trolley, screen, medical waste bags  - Urine container for general examination  - Referral for general urine examination | |
| Initial parameters  - simulator | N/A | |
| Interview | The patient does not understand why she needs to collect urine again. Moreover, she cannot sleep because she is woken up every day first thing in the morning. | |
| Progress of the scenario | 1. Participants enter the room where the patient is located 2. Inform patient that urine is to be collected for examination 3. Making the decision to catheterise the bladder 4. Obtain consent from the patient to perform the procedure 5. Inserting bladder catheter 6. Collection of urine for general examination 7. Removal of catheter 8. Transfer of collected urine to laboratory 9. Documentation of urine collection | |
| Necessary props | Standard patient room equipment, bladder catheterisation kit, urine container, referral for examination | |

Simulation scenario no. 3

| Keep it a secret from the training participants.  Knowledge of the scenario reduces the satisfaction of solving the task | | |
| --- | --- | --- |
| Objectives – to be discussed at debriefing | Technical:  - correct insertion of bladder catheter  - insertion of fluid balance chart | Soft skills:  - communicating with the patient with a depressed mood  - communicating with the team |
| Title of the simulation scenario | Fluid balance in a patient with circulatory failure | |
| Authors | Agata Wojcieszek  Kinga Kołodziej | |
| Contact | [Agata.wojcieszek@uj.edu.pl](mailto:Agata.wojcieszek@uj.edu.pl) | |
| Diagnosis | Insertion of a bladder catheter to guide fluid balance | |
| Case description | Patient Krystyna P. (69 years) is in the internal medicine ward  due to chronic circulatory insufficiency. On the lower limbs and sacro-lumbar region, pasty swellings are noticeable. The patient is incapable of self-service, e.g. unable to walk to the toilet independently. The doctor on duty has ordered a fluid balance (see order sheet).  The patient has a depressed mood. She reflects on the meaning of human life and her impending death. Occasionally, she does not agree to certain treatments because she considers them pointless. | |
| Team composition | Instructor - plays the role of the nervous patient, supervises the scenario  The rest of the team - nursing students who take part in the scenario | |
| Information for participants | - | Team (roles): appoint a team leader. |
| Preparing the mannequin | A female simulator on a hospital bed wearing a long shirt. | |
| Room | Patient room in the internal medicine department | |
| Equipment List | - Permanent urinary bladder catheterisation kit:  sterile disposable catheter (2 pcs), sterile grip tool, sterile disposable gloves (2 pcs), non-sterile disposable gloves, sterile disposable drape, sterile gauze pads (minimum 6 pcs), anaesthetic-slip material e.g. 2% lidocaine, antiseptic fluid e.g. Skinsept Mucosa, urine bag, ampoules with water for injections, 2 sterile needles, sterile syringes 10-20 ml for filling the sealing balloon, kidney bowls, urine bag hanger, buttock pad, treatment tray/cart, screen, medical waste bags  - Fluid balance sheet  - Medical order sheet | |
| Initial parameters  - simulator | 20 breaths/min; SpO2 - 97% Temp 36.7°C; HR: 100 bpm, irregular, well-tensed; RR 140/80 mmHg. | |
| Interview | The patient has been diagnosed with circulatory failure for a year. It is accompanied by symptoms including fatigue, shortness of breath that worsens when lying down and oedema.  Ms Christine wonders aloud about the advisability of bladder catheterisation - ‘there's no point’, ‘I'm going to die anyway’, ‘what's the point?’. | |
| Progress of the scenario | 1. Entering the room where the patient is located  2. Become familiar with the medical order  3. Interview patient  4. Obtain consent to place a bladder catheter due to mandated fluid balance  5. Perform procedure  6. Insertion of fluid balance sheet  7. Documentation of catheter insertion | |
| Necessary props | Standard patient room equipment, permanent bladder catheterisation kit, documentation | |
