# Supplementary material for: Analysis of high-fidelity simulation effects and their connection with educational practices in early nursing education
Source: BMC Nurs. 2025 Apr 24;24:457. doi: 10.1186/s12912-025-03077-x (PMC12023673; doi:10.1186/s12912-025-03077-x)
Supplement: Supplementary file 2 — Supplementary Material 2 [file 12912_2025_3077_MOESM2_ESM.docx]

| Scenario No …………………………………………………………………………………  …………………………………………………………………………………  Instructor ………………………………………………………………………  …………………………………………………………………………………  Group number: ………………………………………………………………..  Date ……………………………………………………………………………  Whether the students: | | Evaluation of the performance of the procedure/activity | | | Moderator's comments: |
| --- | --- | --- | --- | --- | --- |
|  |  | **Correctly** | **Partially correct** | **Incorrectly** |  |
| 1. | have identified the patient |  |  |  |  |
| 2. | initiate a discussion with the patient about the procedure - state the purpose, course and method of the procedure |  |  |  |  |
| 3. | take into account the mood/emotions of the patient/family during the procedure |  |  |  |  |
| 4. | have obtained the patient's consent to perform the procedure |  |  |  |  |
| 5. | have prepared themselves for the procedure |  |  |  |  |
| 6. | have prepared the kit needed to carry out the procedure |  |  |  |  |
| 7. | have prepared the patient environment |  |  |  |  |
| 8. | prepare the patient for the procedure |  |  |  |  |
| 9. | performed a bladder catheterisation procedure |  |  |  |  |
| 10. | tidied up the kit when the activity was over |  |  |  |  |
| 11. | have documented the treatment |  |  |  |  |
| 12. | set up/maintain documentation related to the procedure performed (fluid balance sheets, completion of referral, etc.). |  |  |  |  |
| Additional criteria:  Do students: | | **Correctly** | **Partially correct** | **Incorrectly** |  |
| 13. | communicate as a team |  |  |  |  |
| 14. | observed the principles of aseptics and antisepsis when performing the procedure |  |  |  |  |
